# COVID-19 and Hemoglobinopathies: A Systematic Review of Clinical Presentations, Investigations, and Outcomes

**DOI:** 10.3389/fmed.2021.757510

**Published:** 2021-10-13

**Authors:** Jun Xin Lee, Wei Keong Chieng, Sie Chong Doris Lau, Chai Eng Tan

**Affiliations:** ^1^Department of Pediatrics, Faculty of Medicine, Universiti Kebangsaan Malaysia, Kuala Lumpur, Malaysia; ^2^Department of Family Medicine, Faculty of Medicine, Universiti Kebangsaan Malaysia, Kuala Lumpur, Malaysia

**Keywords:** COVID-19, hemoglobinopathies, sickle cell disease, thalassemia, severe acute respiratory syndrome coronavirus 2, systematic review

## Abstract

This systematic review aimed to provide an overview of the clinical profile and outcome of COVID-19 infection in patients with hemoglobinopathy. The rate of COVID-19 mortality and its predictors were also identified. A systematic search was conducted in accordance with PRISMA guidelines in five electronic databases (PubMed, Scopus, Web of Science, Embase, WHO COVID-19 database) for articles published between 1st December 2019 to 31st October 2020. All articles with laboratory-confirmed COVID-19 cases with underlying hemoglobinopathy were included. Methodological quality was assessed using the Joanna Briggs Institute (JBI) critical appraisal checklists. Thirty-one articles with data on 246 patients with hemoglobinopathy were included in this review. In general, clinical manifestations of COVID-19 infection among patients with hemoglobinopathy were similar to the general population. Vaso-occlusive crisis occurred in 55.6% of sickle cell disease patients with COVID-19 infection. Mortality from COVID-19 infection among patients with hemoglobinopathy was 6.9%. After adjusting for age, gender, types of hemoglobinopathy and oxygen supplementation, respiratory (adj OR = 89.63, 95% CI 2.514–3195.537, *p* = 0.014) and cardiovascular (adj OR = 35.20, 95% CI 1.291–959.526, *p* = 0.035) comorbidities were significant predictors of mortality. Patients with hemoglobinopathy had a higher mortality rate from COVID-19 infection compared to the general population. Those with coexisting cardiovascular or respiratory comorbidities require closer monitoring during the course of illness. More data are needed to allow a better understanding on the clinical impact of COVID-19 infections among patients with hemoglobinopathy.

**Clinical Trial Registration:** https://www.crd.york.ac.uk/prospero/display_record.php?ID=CRD42020218200.

## Introduction

The unprecedented coronavirus disease-2019 (COVID-19) pandemic has not abated since the first-ever reported case in Wuhan, China. The World Health Organization (WHO) declared the outbreak of Public Health Emergency of International Concern on 30th January 2020, and subsequent pandemic on 11th March 2020. According to WHO COVID Dashboard, as of 9th August 2021, COVID-19 has impacted more than 200 million patients globally, with incidence and mortality rates of 2.58 and 2.12%, respectively. The emergence of new variants that are associated with higher severity and mortality has added more burden to the already exhausted health care system.

COVID-19 is known to spread through respiratory droplets, with recent evidence of airborne transmission. Published studies suggested 17.9–30.8% of infected patients may be asymptomatic (1, 2). Symptoms of COVID-19 infection vary from the common presentation of fever, cough, and shortness of breath, to the less common ones such as anosmia, ageusia, and diarrhea (3, 4). Several risk factors were identified to be associated with higher mortality including age more than 65 years old and the presence of chronic diseases such as diabetes mellitus and cardiovascular disease.

Haemoglobinopathy itself is a chronic disease. Patients with hemoglobinopathy are a specific population with special health needs. Cardiopulmonary comorbidities that arise as complications of the disease are one of the main causes of mortality and morbidity in this population (5). Concerns arise whether this group of patients is more susceptible to COVID-19 infection with a more severe course of illness given their immunocompromised state and its many comorbidities. Clinicians need to be aware of the potential differences in how COVID-19 infection manifests in patients with hemoglobinopathies, along with the possible risk factors associated with poorer outcomes. As most evidence is being published as case series or case reports, there is a need to synthesize these findings to guide clinicians in managing COVID-19 infection in patients with hemoglobinopathy.

Hence, this systematic review aims to provide an overview of the clinical profile, including the clinical presentations, laboratory, and radiological findings, as well as the outcome of COVID-19 infection among patients with hemoglobinopathy.

## Materials and Methods

### Search Strategy and Selection Criteria

The study methods were in adherence to the guidelines established by Preferred Reporting Items for Systematic Reviews and Meta-Analyses (PRISMA). The study protocol was registered in the International Prospective Register of Systematic Reviews (PROSPERO) (Protocol number #CRD42020218200).

A systematic search was conducted in the following databases: PubMed, Scopus, Web of Science, Embase, and WHO COVID-19 database. The search terms included were “COVID-19” OR “severe acute respiratory syndrome coronavirus 2” OR “ncov” OR “2019-nCoV” OR “COVID-19” OR “SARS-CoV-2” AND “Hemoglobinopathies” OR “Thalassemia” OR “Anemia, Sickle Cell” OR Hemoglobin C Disease. The last search was performed on 30th October 2020.

Articles that were eligible for review included the following study designs: systematic review, cohort, case-control, cross-sectional, case report, and case series. Only articles published from 1st December 2019 to 31st October 2020, in English which reported laboratory-confirmed COVID-19 cases with underlying hemoglobinopathy were included in this review. In this review, hemoglobinopathies were defined as a heterogeneous group of inherited disorders characterized by structural alterations within the hemoglobin molecule, specifically sickle cell disease and thalassemia. This review included both the adult and pediatric populations. Articles that reported suspected COVID-19 cases without laboratory evidence, *in vitro* studies, animal experiments, or patients without hemoglobinopathy were excluded from this review. Papers that consisted of only an abstract were also excluded.

Outcomes of interest for this study were clinical presentation, laboratory, and radiological findings, and outcomes of COVID-19 infection among patients with hemoglobinopathies.

### Data Collection and Risk of Bias Assessment

The studies extracted from the searches were identified by two independent reviewers. Citation records were managed with EndNote(R). Duplicate citations were deleted and the records were exported to an Excel sheet. Each citation was screened based on titles, abstracts, and keywords. Reasons for excluding citations were recorded. The full articles fulfilling the inclusion criteria were retrieved for review. A third investigator was consulted to resolve differences of opinion at any phase. Data retrieved from each article was cross-checked by at least two independent investigators.

The Excel data extraction form recorded the following information: author/s, study title, study design, country of study, year of publication, digital object identifier (DOI), sample size, comorbidities, clinical signs and symptoms, laboratory findings, imaging findings, outcomes.

The quality of studies included was appraised using the Joanna Briggs Institute (JBI) critical appraisal tools for the respective study designs (6). The studies were further classified into poor, moderate, or high quality based on selected criteria that would provide sufficient information for the purpose of this study (Supplementary Material). The risk of bias was assessed independently by two investigators and any discrepancies in opinions were resolved by a third investigator.

### Operational Definitions

Symptoms were considered present if they occurred at any time from presentation to discharge. Duration of symptoms was presented in days.

All laboratory data results were categorized into high, normal, or low, according to the local laboratory reference values in the respective articles. This was done taking into consideration that different laboratories would have different reference ranges. For articles that reported mean values for multiple samples, the researcher attempted to email the original authors for their raw data for further analysis. In the event that raw data was not available, they were considered as missing data.

Radiological findings were categorized based on the descriptive changes reported by the authors: normal, ground glass opacity, consolidation, combined ground glass opacity/consolidation and others.

Clinical staging for COVID severity was based on the National Institute of Health guidelines category 1 for asymptomatic presentation, category 2 for mild illness (mild systemic and respiratory symptoms with no clinical evidence of lower respiratory involvement), category 3 for moderate illness (clinical signs and symptoms of lower respiratory involvement with oxygen saturation of or more than 95% on room air at sea level), category 4 for severe illness (lower respiratory involvement with oxygen saturation of <95% on room air at sea level) and category 5 for critical illness (presence of acute respiratory distress syndrome, septic shock or multiorgan involvement) (7).

### Statistical Analysis

Descriptive statistics were reported using frequencies, percentages, and ranges. The proportion ratios and prevalence rates were also determined. Simple logistic regression was done to determine the crude odds ratio for various comorbidities and COVID-19 mortality. Comorbidities with *p*-value of <0.25 were included in the model for binary logistic regression. Binary logistic regression was conducted to determine the independent associations between selected predictors with COVID-19 mortality, with adjustment for age, gender, and need for oxygen supplementation. All statistical analyses were done using Statistical Package for the Social Sciences (SPSS) version 26. The α for statistical significance was set at 0.05.

## Results

**Figure 1:**
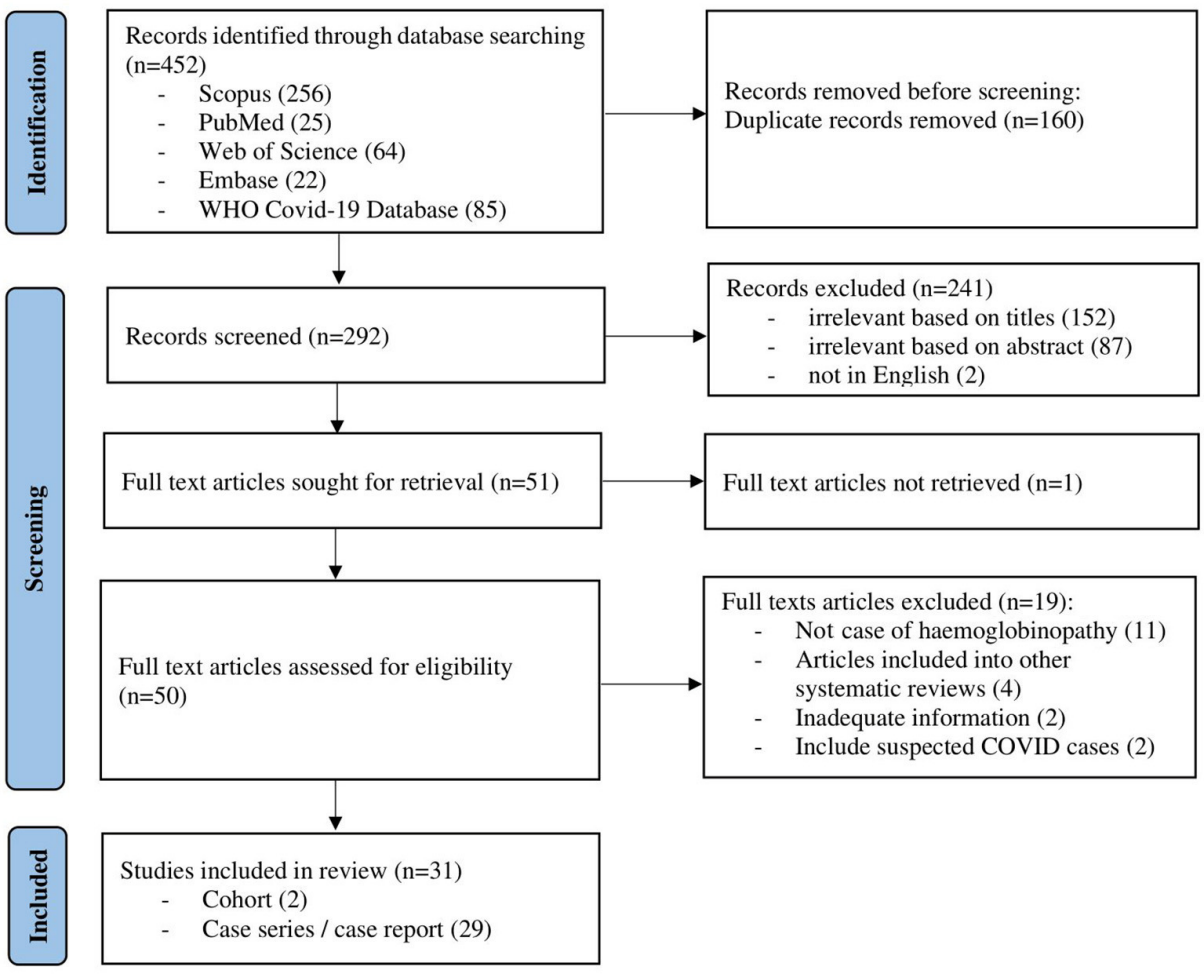
Flow chart of study selection process (flow chart adapted from PRISMA).

The initial search of the electronic databases yielded 452 articles. Manual searching through the references of these articles yielded no additional eligible articles. After removing 160 duplicates, the titles and abstracts for 292 articles were screened. Following this, 50 full-text articles were retrieved with the full text of 1 article being unable to be retrieved. Finally, out of the 50 eligible full-text articles, 31 articles were included in this review (Figure 1). Of these, 2 were retrospective cohort studies, 13 were case series and 16 were case reports (Table 1). As the majority of the articles were case reports and case series, the level of evidence was low. The methodological quality for all of the studies is described in the supplementary data (Supplementary Material).

**Table 1:** Characteristics of studies included.

**No**	**First Author**	**Study Type**	**Country**	**N**	**Age (Years)**	**Adult, ***n*****	**Male, ***n*****	**Types of Hemoglobinopathy**		**Mortality, ***n*****
								**SCD, n**	**Thal, n**	
1	Albagshi M (8)	Case Series	Saudi Arabia	2	37	2	1	2	0	0
2	Al-Hebshi A (9)	Case Series	Saudi Arabia	3	42.667	1	1	3	0	0
3	Allison D (10)	Case Report	United States	1	27	1	1	1	0	0
4	Appiah-Kubi A (11)	Case Series	United States	7	14.286	2	2	7	0	0
5	Arlet JB (12)	Case Series	France	83	Adult: 33.5 (median) Child: 12.0 (median)	66	38	83	0	2
6	Balanchivadze N (13)	Case Series	United States	24	52.9	24	6	24	0	1
7	Beerkens F (14)	Case Report	United States	1	21	1	1	1	0	0
8	Dagalakis U (15)	Case Report	United States	1	0.5	0	1	1	0	0
9	De Luna G (16)	Case Report	France	1	45	1	1	1	0	0
10	De Sanctis V (17)	Case Series	Multi-nation^§^	13	33.7	12	4	3	10	1
11	Ershler WB (18)	Case Report	United States	1	39	1	0	1	0	0
12	Fronza M (19)	Case Report	Italy	1	44	1	0	1	0	0
13	Heilbronner (20)	Case Series	France	4	14.55	0	1	4	0	0
14	Hussain FA (21)	Case Series	United States	4	33	4	2	4	0	0
15	Jacob S (22)	Case Study	United States	1	2.75	0	1	1	0	0
16	Justino (23)	Case Study	Brazil	1	35	1	0	1	0	0
17	Karimi M (24)	Cohort	Iran	15	36	15	7	0	15	4
18	Karimi M (25)	Cohort	Iran	43	35.3	42	22	0	43	8
19	Marhaeni W (26)	Case Report	Indonesia	1	17	0	0	0	1	0
20	Marziali M (27)	Case Report	Italy	1	46	1	1	0	1	0
21	Morrone KA (28)	Case Series	United States	8	16	2	4	8	0	0
22	Motta I (29)	Case Series	Italy	11	44.27	11	5	0	11	0
23	Nur E (30)	Case Series	Netherlands	2	22	2	1	2	0	0
24	Odievre MH (31)	Case Report	Netherlands	1	16	0	0	1	0	0
25	Okar L (32)	Case Report	Qatar	1	25	1	0	0	1	0
26	Pinto VM (33)	Case Report	Italy	1	57	1	1	0	1	0
27	Sasi S (34)	Case Report	Qatar	1	26	1	1	0	1*	0
28	Sheha D (35)	Case Report	Egypt	1	22	1	0	1	0	0
29	Stochino C (36)	Case Series	Italy	1	20	1	0	1	0	0
30	Subarna C (37)	Case Series	United Kingdom	10	38.25	10	3	10	0	1
31	Verdiyeva N (38)	Case Report	Russia	1	24	1	1	1	0	0

*SCD, sickle cell disease; Thal, thalassemia; N, number*.

§ *Turkey, Italy, Cyprus, Iran, Oman, Azerbaijan*.

* *Hemoglobin D disease*.

Overall, a total of 246 patients with hemoglobinopathies were reported to have COVID-19 infection. The patients' age ranged from 0.5 to 61 years old, with the majority (83.7%) being adults (age above 18 years old). Out of this, 140 (56.9%) had sickle cell anemia, 22 (8.9%) had sickle cell trait, 68 (27.6%) transfusion-dependent thalassemia and 16 (6.5%) non-transfusion-dependent thalassemia. Two-third (64.6%) of the patients had at least one underlying comorbidity and 22.4% had undergone splenectomy (Table 2). Among the sickle cell anemia patients, 42.6% had a history of vaso-occlusive crisis.

**Table 2:** Underlying comorbidities of the study population.

**Underlying Medical Conditions**	**Thal, ***n*** (%)**	**SCD, ***n*** (%)**
	**[***N*** = 84]**	**[***N*** = 162]**
None	23 (27.4)	64 (39.5)
**Hematology**		
Vaso-occlusive crisis	N/A	69 (42.6)
Splenectomy	49 (58.3)	6 (3.7)
**Cardiology**		
Hypertension	2 (2.4)	12 (7.4)
Pulmonary hypertension	12 (14.3)	0 (0)
Heart Failure	8 (9.5)	0 (0)
Cardiomyopathy	5 (6.0)	1 (0.6)
Arrhythmia	1 (1.2)	1 (0.6)
Not specified	1 (1.2)	0 (0)
**Respiratory**		
Asthma	2 (2.4)	9 (5.6)
Pulmonary embolism	0 (0)	3 (1.9)
Obstructive sleep apnea	1 (1.2)	1 (0.6)
Chronic obstructive pulmonary disease	0 (0)	1 (0.6)
Sarcoidosis	1 (1.2)	0 (0)
Concurrent pulmonary tuberculosis	0 (0)	1 (0.6)
Not specified	1 (1.2)	0 (0)
**Endocrinology**		
Diabetes mellitus	19 (22.6)	10 (6.2)
Hypogonadism	20 (23.8)	0 (0)
Obesity	1 (1.2)	13 (8.0)
Hypothyroidism	9 (10.7)	0 (0)
Hypoparathyroidism	6 (7.1)	0 (0)
Growth failure	3 (3.6)	0 (0)
**Orthopedics**		
Osteoporosis	41 (48.8)	0 (0)
Avascular necrosis	0 (0)	2 (1.2)
**Hepatobiliary**		
Chronic liver disease	14 (16.7)	0 (0)
Hepatitis	10 (11.9)	0 (0)
Gallstone disease	1 (1.2)	0 (0)
**Nephrology**		
Chronic kidney disease / end stage renal failure	8 (9.5)	2 (1.2)
**Vascular**		
Venous thromboembolism	0 (0)	7 (4.3)
Recurrent leg ulcer	0 (0)	2 (1.2)
**Neurology**		
Stroke	0 (0)	6 (3.7)
Transient ischemic attack	0 (0)	1 (0.6)
Moya Moya syndrome	0 (0)	1 (0.6)
**Oncology**		
Non-Hodgkin lymphoma	1 (1.2)	0 (0)
Acute lymphoid leukemia	1 (1.2)	0 (0)
Not specified	0 (0)	5 (3.1)
**Ophthalmology**		
Retinopathy	0 (0)	2 (1.2)
**Psychiatry**		
Not specified	0 (0)	1 (0.6)

*SCD, sickle cell disease; Thal, thalassemia; N, number; N/A, not applicable*.

Table 3 summarized the reported clinical symptoms, laboratory markers, and radiological findings for COVID-19 infection in these patients. Twenty-nine (35.8%) patients had severe COVID-19 (Stage 4 or 5). The three most common presenting symptoms were fever (69.2%), vaso-occlusive crises (55.6%), and cough (54.2%). A small number of patients presented with mild non-respiratory symptoms such as gastrointestinal symptoms, conjunctivitis, and anorexia. Eight (6.7%) adult COVID-19 patients were detected through mass screening and were asymptomatic. Half (55.8%) of the patients had normal SpO_2_ during admission. Complete blood counts and C-reactive protein (CRP) were the most commonly reported laboratory results (Table 3). Overall, 79.5% (*n* = 105) of patients were anemic at presentation and 50.7% (*n* = 34) had leukocytosis. Radiographic findings were only reported in a small number of patients (*n* = 45) with 64.1% and 76.4% having radiological features of COVID-19 in chest x-ray and computed tomography, respectively.

**Table 3:** Summary of clinical presentations, laboratory investigations and radiological imaging of the study population.

**Parameters**	**Thal, ***n*** (%)**	**SCD, ***n*** (%)**
**Clinical symptoms**	***n*** **= 41**	***n*** **= 79**
Asymptomatic	1 (2.4)	7 (8.9)
**Systemic symptoms**		
Fever	31 (75.6)	52 (65.8)
Vaso-occlusive crisis	N/A	90 (55.6)
Fatigue	16 (39.0)	2 (2.5)
Anorexia	7 (17.1)	7 (8.9)
**Respiratory**		
Cough	30 (73.2)	35 (44.3)
Shortness of breath	13 (31.7)	23 (29.1)
Sore throat	10 (24.4)	2 (2.5)
Nasal symptoms (rhinorrhea, sneezing, sinusitis)	6 (14.6)	3 (3.8)
**Gastrointestinal**		
Nausea, vomiting, diarrhea	13 (31.7)	14 (17.7)
**Musculoskeletal**		
Myalgia	4 (9.8)	21 (26.6)
**Neurological**		
Headache	11 (26.8)	9 (11.4)
Anosmia	12 (29.3)	3 (3.8)
Ageusia	7 (17.1)	2 (2.5)
**Ophthalmology**		
Conjunctivitis	3 (7.3)	0 (0)
**SpO**_**2**_	***n*** **= 41**	***n*** **= 79**
≥95%	15 (36.6)	52 (65.8)
<95%	26 (63.4)	27 (34.2)
Laboratory investigations^*^		
**Hemoglobin**	***n*** **= 68**	***n*** **= 64**
Normal	6 (8.8)	21 (32.8)
Low	62 (91.2)	43 (67.2)
**White cell count**	***n*** **= 42**	***n*** **= 25**
Normal	17 (40.5)	14 (56.0)
High	23 (54.8)	11 (44.0)
Low	2 (4.8)	0 (0)
**Lymphocyte count**	***n*** **= 14**	***n*** **= 30**
Normal	12 (85.7)	16 (53.3)
High	1 (7.1)	2 (6.7)
Low	1 (7.1)	12 (40.0)
**Neutrophil count**	***n*** **= 4**	***n*** **= 1**
Normal	2 (50.0)	0 (0)
High	1 (25.0)	1 (100.0)
Low	1 (25.0)	0 (0)
**Platelet**	***n*** **= 48**	***n*** **= 46**
Normal	27 (55.1)	31 (67.4)
High	21 (42.9)	8 (17.4)
Low	0 (0)	7 (15.2)
**C-Reactive protein**	***n*** **= 14**	***n*** **= 32**
Normal	8 (57.1)	8 (25.0)
High	6 (42.9)	24 (75.0)
**D-dimer**	***n*** **= 4**	***n*** **= 17**
Normal	1 (25.0)	1 (5.9)
High	3 (75.0)	16 (94.1)
**Lactate dehydrogenase**	***n*** **= 11**	***n*** **= 10**
Normal	7 (63.6)	0 (0)
High	4 (36.4)	10 (100.0)
**Erythrocyte sedimentation rate**	***n*** **= 10**	***n*** **= 4**
Normal	7 (70.0)	0 (0)
High	3 (30.0)	4 (100.0)
**Procalcitonin**	***n*** **= 1**	***n*** **= 5**
Normal	1 (100.0)	1 (20.0)
High	0 (0)	4 (80.0)
**Fibrinogen**	***n*** **= 1**	***n*** **= 4**
High	1 (100.0)	4 (100.0)
**Radiological findings**		
**Chest X-ray**	***n*** **= 12**	***n*** **= 27**
Normal	4 (33.3)	10 (37.0)
Ground glass opacity	0 (0)	2 (7.4)
Consolidation	1 (8.3)	9 (33.3)
Combined ground glass opacity and consolidation	0 (0)	1 (3.8)
Others^§^	0 (0)	5 (18.5)
Not specified	7 (58.4)	0 (0)
**Computed Tomography thorax**	***n*** **= 8**	***n*** **= 9**
Normal	3 (37.5)	1 (11.1)
Ground glass opacity	1 (12.5)	0 (0)
Consolidation	0 (0)	2 (22.2)
Combined ground glass and consolidation	0 (0)	4 (44.5)
Others^†^	1 (12.5)	2 (22.2)
Not specified	3 (37.5)	0 (0)
**COVID-19 staging^‡^**	***n*** **= 26**	***n*** **= 55**
1	0 (0)	8 (14.5)
2	7 (26.9)	22 (40.0)
3	9 (34.6)	6 (10.9)
4	10 (38.5)	10 (18.2)
5	0 (0)	9 (16.4)

*SCD, sickle cell disease; Thal, thalassemia; N, number; N/A, not applicable*.

* *Categorized into high, normal or low according to the articles' local laboratory reference values*.

§ *Described changes such as reticular opacity, atelectasis, perihilar streaking, interstitial or alveolar infiltration*.

† *Described changes such as interstitial or alveolar infiltration, halo sign*.

‡ *Staging based on NIH guidelines (7)*.

Twenty-eight articles reported treatment given to the patients (*n* = 93) (Table 4). Antibiotics (67.3%) and hydroxychloroquine (58.2%) were the more commonly administered drugs. The majority (82.4%) of patients required hospital admission with about 29.3% of them requiring supplemental oxygen (either non-invasive or invasive), and 31.9% required blood or exchange transfusion. There were 17 (6.9%) deaths reported; out of those, 13 (76.5%) were thalassemia patients while the remaining were patients with SCD.

**Table 4:** Summary of treatments received and outcome of study population.

**Parameters**	**Thal, ***n*** (%)**	**SCD, ***n*** (%)**
**Hospitalization**	***n*** **= 26**	***n*** **= 162**
Yes	16 (61.5)	139 (85.8)
No	10 (38.5)	23 (14.2)
**Treatments**	***n*** **= 12**	***n*** **= 43**
Hydroxychloroquine	6 (50.0)	26 (60.5)
Antibiotics*	9 (75.5)	28 (65.1)
Anti-viral	5 (41.7)	0 (0)
**Disease modifying agent**		
Tocilizumab	1 (8.3)	4 (9.3)
Anakinra	1 (8.3)	3 (7.0)
Glucocorticoid	1 (8.3)	3 (7.0)
**Oxygen support**	***n*** **= 26**	***n*** **= 162**
No oxygen required	15 (57.7)	118 (72.8)
Non-invasive	11 (42.3)	33 (20.4)
Invasive	0 (0)	11 (6.8)
**Transfusion**	***n*** **= 26**	***n*** **= 162**
No transfusion required	21 (80.8)	107 (66.0)
Packed cell transfusion	5 (19.2)	45 (27.8)
Exchange transfusion	N/A	4 (2.5)
Packed cell and exchange transfusion	N/A	6 (3.7)
**Treatment outcome**	***n*** **= 84**	***n*** **= 162**
Recovered	71 (84.5)	158 (97.5)
Death	13 (15.5)	4 (2.5)

*SCD, sickle cell disease; Thal, thalassemia; n, number; N/A, not applicable*.

* *Types of antibiotics used includes cephalosporin, macrolide, fluoroquinolone, beta-lactamase and tetracycline*.

Binary logistic regression analysis was done to determine the independent association between comorbidities and mortality, adjusting for age, gender, types of hemoglobinopathy, and oxygen supplementation. The model predicted between 8.0 and 36.5% variance in the outcome and was able to correctly predict 97.3% of mortality outcomes. The presence of respiratory and cardiovascular comorbidities was independently associated with mortality in patients with underlying hemoglobinopathies who were infected with COVID-19 (Table 5). Respiratory comorbidities were associated with 89.63 times risk of death compared to those without respiratory comorbidities (adj OR = 89.63, 95% CI 2.514–3195.537, *p* = 0.014). Cardiovascular comorbidities were associated with 35.20 times risk of death compared to those without (adj OR = 35.20, 95% CI 1.291–959.526, *p* = 0.035).

**Table 5:** Predictors of COVID-19 mortality in patients with hemoglobinopathy.

**Variables**	**Simple logistic regression**			**Binary logistic regression**		
	**Crude odds ratio**	**95% CI**	***p*** **-value**	**Adjusted odds ratio**	**95% CI**	***p*** **-value**
Gender	2.015	(0.741, 5.483)	0.170	1.225	(0.086, 17.391)	0.881
(female vs. male)				
Age	1.492	(0.328, 6.795)	0.605	0.171	(0.009, 3.179)	0.237
(pediatric vs. adult)						
Types of hemoglobinopathy	7.232	(2.278, 22.958)	0.001^*^	14.612	(0.425, 502.262)	0.137
(Thal vs. SCD)						
Respiratory comorbidity	1.902	(0.398, 9.103)	0.421	89.625	(2.514, 3195.537)	0.014^*^
(no vs. yes)						
Cardiovascular comorbidity	4.081	(1.396, 11.930)	0.010^*^	35.199	(1.291, 959.526)	0.035^*^
(no vs. yes)						
Splenectomy	3.442	(1.260, 9.403)	0.016^*^	<0.001	–	0.998
(no vs. yes)						
Diabetes mellitus	2.511	(0.760, 8.296)	0.131	<0.001	–	0.998
(no vs. yes)						
Oxygen requirement	3.779	(0.614, 23.271)	0.152	8.912	(0.674, 117.824)	0.097
(no vs. yes)						

*CI, confidence interval; Thal, thalassemia; SCD, sickle cell disease*.

*Simple logistic regression was performed for all comorbidities vs. mortality as an outcome. Comorbidities with p-value of <0.25 for simple logistic regression were included in the model for binary logistic regression*.

* *Indicate statistically significant p < 0.05*.

## Discussions

Our review found that in terms of severity, more patients with hemoglobinopathy (35.8%) had severe COVID-19 infection compared to the general population (11.1–19.1%) (39, 40). This could explain the higher mortality due to COVID-19 in this review (6.9%) compared to the general population (2.2–5.0%) (3, 39, 40). The mortality of 6.9% in patients with hemoglobinopathy was higher than reported COVID-19 mortality in patients with chronic kidney disease (1.54%) (41). However, it was lower compared to other immunocompromised conditions such as HIV infection (12.65%) (42) and malignancy (25.6%) (43). Majority of the published cases in this review was from high-income countries (25 articles; 17 thalassemia and 159 SCD patients) with four (2.5%) deaths involving SCD patients reported. In contrast, most of the thalassemia cases reported were from the low-middle-income countries (5 articles; 63 thalassemia patients) of which, there were 13 (20.6%) deaths. As the type of hemoglobinopathy is not a significant predictor of mortality in our logistic regression, we postulate that the higher mortality rate among the thalassemia patients could be attributed to the low health care system capacity in the low-middle-income countries.

This review also found that the presence of respiratory and cardiovascular comorbidities were independent predictors of mortality in COVID-19 infection. COVID-19 infection can lead to multi-system inflammation, resulting in myocardial injuries such as myocarditis, arrhythmia, acute coronary syndrome, and venous thromboembolism (44). COVID-19 related myocardial injury may further aggravate the burden of patients with underlying cardiovascular comorbidities, thereby putting them at higher risk of death (44). COVID-19 infection is primarily a viral respiratory illness. In chronic pulmonary disease, alteration of the pulmonary structures may result in a more severe COVID-19 infection. Up-regulation of ACE-2 expression in COPD, and delayed innate antiviral immune response in asthma (45) are mechanisms that possibly explain the higher risk of severe COVID-19 infection and mortality among those with respiratory comorbidities (40).

Several studies have found that SCD patients with heart and lung comorbidities were at higher risk of severe COVID-19 infections (46, 47). Similarly, the mortality rate among thalassemia patients with cardiovascular comorbidities was significantly higher when compared to its counterpart without underlying cardiovascular disease (48). Thalassemia patients are expected to be at higher risk of cardiovascular comorbidities compared to SCD patients. This is due to the higher risk of cardiomyopathy associated with iron overload compared to patients with sickle cell disease (49). Cardiomyopathy is also the leading cause of mortality and morbidity in thalassemia patients (50, 51). However, this excess risk of COVID-19 mortality due to the presence of cardiovascular comorbidity remained statistically significant despite statistically controlling for the type of hemoglobinopathy. Due to the relatively small number of samples, the precision of the adjusted OR was affected, resulting in an extremely wide confidence interval. As more evidence is being generated, a more precise estimate of the true adjusted odds ratio can be determined.

In this review, the clinical presentations of COVID-19 infection among patients with hemoglobinopathy were almost similar to the general population, with fever and cough being reported as the most common symptoms (52, 53). COVID-19 infection may also lead to hypoxia, triggering vaso-occlusive crisis, making this one of the common manifestations seen in patients with SCD. Anemia, leukocytosis, lymphopenia, thrombocytosis, and raised CRP were among the abnormal laboratory changes reported (54, 55). These are among the common non-specific findings in the presence of infections. The treatment and management of COVID-19 in these patients were similar to that of COVID-19 patients in the general population (56–58). Exchange transfusion was only required in a small number of patients with sickle cell disease who presented with vaso-occlusive crises and severe COVID-19 infections (n=10, 6.2%). Meanwhile, blood transfusion was generally required when patients with hemoglobinopathy had anemia during presentation (mean hemoglobin 8.2 ± 1.6 g/dL). As the data available was limited, we were unable to determine whether the requirement for transfusion was influenced by the severity of COVID-19 infection and oxygen requirement. None of the articles in this review reported on the impact of COVID-19 pandemic on blood transfusion services at their center, and whether this indirectly affect the management as well as the outcome of patients with hemoglobinopathy when they were admitted for COVID-19 infection. Two studies conducted in Eastern Mediterranean reported no interruption on blood transfusion services for patients with hemoglobinopathy despite the pandemic (59, 60). However, more data is needed especially from the low- and middle-income countries with regards to this matter.

This systematic review summarized the clinical manifestations, investigations, and outcomes of COVID-19 in the hemoglobinopathy population. One of the limitations was that studies included in this review were published during the early phase of COVID-19 pandemic, thus most of them were case reports or case series with low levels of evidence. The small sample size also discouraged further analysis based on types of hemoglobinopathy. Furthermore, not all of the studies reported their laboratory and radiological findings, hence a meta-analysis was not feasible to conclusively determine the association between laboratory markers and disease outcomes. Another limitation in our review was that the radiological findings were categorized based on the descriptive changes reported by the authors. As both COVID-19 pneumonia and acute chest syndrome in SCD patients may have similar findings (consolidation, ground glass opacity, and atelectasis), we were unable to differentiate what causes these radiological changes. The outcome of the study population reported in this systematic review was limited to mortality rate as no follow-up data were available with regards to long term effects of COVID-19 infection to patients with hemoglobinopathy. Finally, the actual number of COVID-19 cases or deaths among patients with hemoglobinopathy may be underreported especially in developing or under-developed nations, given limitations of resources for testing. The development of an authoritative international registry to capture the data on COVID-19 infections among patients with hemoglobinopathy will allow a more accurate and impactful analysis.

## Conclusion

This review has shown that there is no difference in terms of clinical manifestations, laboratory and imaging findings for COVID-19 infections among patients with hemoglobinopathy compared to the general population. There is higher COVID-19 mortality among patients with hemoglobinopathy compared to the general population. Clinicians who manage COVID-19 infections in patients with underlying hemoglobinopathy should therefore exercise greater caution, especially in the presence of coexisting cardiovascular or respiratory comorbidities. Further large-scale, longitudinal studies are needed to evaluate the impact and long-term morbidity of COVID-19 infection on patients with hemoglobinopathy.

## Data Availability Statement

The original contributions presented in the study are included in the article/Supplementary Material, further inquiries can be directed to the corresponding author/s.

## Author Contributions

JL, WC, CT, and SL contributed to the conception and design of the study. JL and WC conducted the systematic search of the study. JL, WC, and CT contributed to the data analysis. All authors contributed to the drafting and revising the article and gave final approval of the version for publication.

## Conflict of Interest

The authors declare that the research was conducted in the absence of any commercial or financial relationships that could be construed as a potential conflict of interest.

## Publisher's Note

All claims expressed in this article are solely those of the authors and do not necessarily represent those of their affiliated organizations, or those of the publisher, the editors and the reviewers. Any product that may be evaluated in this article, or claim that may be made by its manufacturer, is not guaranteed or endorsed by the publisher.
