# Supplementary material for: COVID-19 and Hemoglobinopathies: A Systematic Review of Clinical Presentations, Investigations, and Outcomes
Source: Front Med (Lausanne). 2021 Oct 13;8:757510. doi: 10.3389/fmed.2021.757510 (PMC8549676; doi:10.3389/fmed.2021.757510)
Supplement: Supplementary file 1 [file Data_Sheet_1.docx]

**Supplementary Table 1** *│*Methodological quality for cohort studies.

|  | JBI 1* | JBI 2* | JBI 3* | JBI 4* | JBI 5 | JBI 6* | JBI 7* | JBI 8* | JBI 9* | JBI 10 | JBI 11 | Quality |
| --- | --- | --- | --- | --- | --- | --- | --- | --- | --- | --- | --- | --- |
| Karimi M et al  (Mortality) | Yes | Yes | Yes | Yes | No | Yes | Yes | Yes | Yes | N/A | Yes | Good |
| Karimi M et al  (Severity) | Yes | Yes | Yes | Yes | No | Yes | No | Yes | Yes | N/A | Yes | Mod |

*JBI criteria considered essential for this review (containing information to be reviewed)

Remarks:

1. Fulfill all essential criteria - Good

2. Up to 2 essential criteria were reported but not clearly explained - Moderate

3. Any essential criteria not reported at all or more than 2 essential criteria reported but not clearly explained - Poor

**Supplementary Table 2** *│*Methodological quality for case series.

|  | JBI  1* | JBI 2* | JBI  3* | JBI  4 | JBI  5 | JBI 6* | JBI  7* | JBI  8* | JBI  9 | JBI 10 | Quality |
| --- | --- | --- | --- | --- | --- | --- | --- | --- | --- | --- | --- |
| Albagashi M et al | Unclear | Yes | Yes | No | No | Yes | Yes | Unclear | Unclear | N/A | Mod |
| Abdulqader Al- Hebshi et al | Unclear | Yes | Yes | No | No | Yes | Yes | Unclear | Yes | N/A | Mod |
| Appiah-Kubi A et al | Yes | Yes | Yes | Yes | Yes | Yes | Yes | No | Unclear | N/A | Poor |
| Arlet JB et al | Yes | Yes | Unclear | Yes | Unclear | Yes | Yes | Yes | Unclear | Yes | Mod |
| Balanchivadze N et al | Yes | Yes | Yes | Yes | Yes | Yes | Yes | Yes | Yes | N/A | Good |
| Heilbronner C et al | Yes | Yes | Yes | Yes | Unclear | Yes | Unclear | Yes | Yes | N/A | Mod |
| Hussain FA et al | No | Yes | Unclear | No | No | Yes | Yes | Yes | No | N/A | Poor |
| Morrone KA et al | Yes | Yes | Yes | Yes | Yes | Yes | Yes | Yes | Yes | Yes | Good |
| Motta I et al | Yes | Yes | Unclear | Yes | Yes | Yes | Yes | Yes | Yes | N/A | Mod |
| Nur E et al | No | Yes | Yes | No | No | Yes | Yes | Unclear | No | N/A | Poor |
| Stochino C et al | Yes | Yes | Yes | Yes | Yes | Yes | Unclear | Unclear | Yes | N/A | Mod |
| Subama C et al | Unclear | Yes | Yes | Yes | No | Yes | Unclear | Yes | Yes | Yes | Mod |

*JBI criteria considered essential for this review (containing information to be reviewed)

Remarks:

1.Fulfill all essential criteria - Good

2.Up to 2 essential criteria were reported but not clearly explained - Moderate

3.Any essential criteria not reported at all or more than 2 essential criteria reported but not clearly explained - Poor

**Supplementary Table 3** *│*Methodological quality for case reports.

|  | JBI 1* | JBI 2* | JBI 3* | JBI 4* | JBI 5* | JBI 6* | JBI 7 | JBI 8 | Quality |
| --- | --- | --- | --- | --- | --- | --- | --- | --- | --- |
| Allison D et al | Yes | Yes | Yes | Unclear | Yes | Yes | Yes | Yes | Mod |
| Beerkens F et al | Yes | Yes | Yes | Yes | Unclear | Yes | No | Yes | Mod |
| Dagalakis U et al | Yes | Unclear | Yes | Yes | Unclear | No | No | Yes | Poor |
| De Luna G et al | Yes | Yes | Yes | Yes | Yes | Yes | No | Yes | Good |
| Ershler WB et al | Yes | No | Unclear | Yes | Yes | Yes | No | Yes | Mod |
| Fronza M et al | Yes | Unclear | Yes | Yes | Unclear | No | No | Yes | Poor |
| Jacob S et al | Yes | Yes | Yes | Yes | Unclear | Yes | No | Yes | Mod |
| Justino CC et al | Yes | Yes | Yes | Yes | Unclear | Yes | No | Yes | Mod |
| Marhaeni W et al | Yes | Yes | Yes | Yes | Yes | No | No | Yes | Mod |
| Marziali M et al | Yes | Yes | Yes | Yes | Unclear | Yes | Yes | Yes | Mod |
| Odievre MH et al | Yes | Yes | Yes | Yes | Unclear | Yes | Yes | Yes | Mod |
| Okar L et al | Yes | Yes | Yes | Yes | Yes | Unclear | No | Yes | Mod |
| Pinto VM et al | Yes | Yes | Yes | Unclear | Yes | Yes | Yes | Yes | Mod |
| Sasi S et al | Yes | Yes | Yes | Yes | Yes | Yes | No | Yes | Good |
| Sheha D et al | Yes | Unclear | Yes | Yes | Unclear | Yes | Yes | Yes | Mod |
| Verdiyeva N et al | Yes | Unclear | Yes | Yes | Unclear | Yes | No | Unclear | Mod |

*JBI criteria considered essential for this review (containing information to be reviewed)

Remarks:

1. Fulfill all essential criteria - Good

2. Up to 2 essential criteria were reported but not clearly explained - Moderate

3. Any essential criteria not reported at all or more than 2 essential criteria reported but not clearly explained - Poor
